# Ocular surface reconstruction in limbal stem cell deficiency

**Published:** 2016

**Authors:** Gheorghe Alina, Pop Monica, Mrini Fildis, Barac Ramona, Vargau Iulia

**Affiliations:** * Clinical Emergency Eye Hospital, Bucharest, Romania

**Keywords:** amniotic membrane, stem cell, allograft, autograft, Goblet cell, ocular surface reconstruction

## Abstract

The purpose of our review was to familiarize the readers with the new concepts in ocular surface diseases and reconstruction. Limbal stem cell deficiency is characterized by the progressive invasion of conjunctival epithelial cells onto the cornea, superficial vascularisation, destruction of the corneal basement membrane, and chronic inflammatory cell infiltration. Depending on the severity of the disease and the time passed from the primary injury amniotic membrane transplantation, keratolimbal allograft and autograft are the available treatments hoping that, in the nearest future, stem cell transplantation and tissue engineering will become the usual therapeutic choices.

**Abbreviations:** LSCD = limbal stem cell deficiency; AMT = amniotic membrane transplant; OSR = ocular surface reconstruction; AM = amniotic membrane

## Introduction

For many years, scientists have been searching for ways to accelerate the healing of the wound and to treat diseases that, until now, have been fatal.

At present, regenerative medicine represents a day reality. New developments have defined new concepts. Ocular surface concept, a concept that refers to the “conjunctiva-limbus- cornea” anatomical and functional group is one of the modern research studies of the ocular surface diseases mechanisms and also an important point of treatment through biological and artificial regeneration [**1**].

These components of ocular surface are essential for the vision and the integrity of the eye [**2**]. In the past, patients with severe ocular surface damage were sentenced to blindness, but now, new research and progress changed the therapeutic approaches by offering new treatment possibilities. New technologies based on embryonic stem cells, tissue engineering, amniotic membrane transplant give a real hope in restoring and maintaining a good vision.

## Pathogenesis

Research made us better understand the physiopathology, define new diagnostic entities and new treatments. Ocular surface reconstruction has recently become a common methodology in the regenerative treatment of severe ocular surface disease. The challenge in this field was motivated by the necessity to find a cure for patients as mentioned above, affected by severe and difficult to treat diseases that damage the integrity of the ocular surface. The most important breakthrough in OSR came when the limbus was identified as the anatomic location of the corneal epithelial stem cells, which led to the development of various effective techniques of limbal stem cell transplantation [**2**].

When the pathologic insults destroy the stem cell, which contains the limbal epithelium, the corneal surface invariably heals with the conjunctival epithelial ingrowth (conjunctivalization), neovascularization, chronic inflammation, and recurrent or persistent corneal epithelial defects. These pathologic signs constitute the newly established disease called limbal (stem cell) deficiency [**3**].

## Diagnosis

Patients suffering from LSCD complain of photophobia and reduced vision as a result of recurrent or persistent corneal epithelial defects [**4**]. In day-to-day practice, the diagnosis of LSCD is clinically made by the loss of limbal palisade of Vogt [**5**] but the most accurate diagnosis is the impression cytology. First used by Tseng and co.[**6**], impression cytology diagnoses limbal deficiency if the conjunctival goblet cells are found on the corneal surface. Of course, depending on the severity of LSCD, impression cytology shows conjunctivalization of the cornea, presence of mucin 1 on the corneal surface and the absence of keratin 12 (as seen in the normal ocular surface) [**7**,**8**]. Histopathologically, LSCD is characterized by a progressive invasion of the conjunctival epithelial cells onto the cornea, superficial vascularisation, destruction of the corneal basement membrane, and chronic inflammatory cell infiltration. These pathological changes explain why corneas characterized by LSCD are not good candidates for conventional keratoplasty [**4**].

## Limbal stem cell deficiency

Limbal stem cell deficiency is characterized by a loss or deficiency of stem cells that are vital for the re-population of the corneal epithelium. Davanger and Evensen [**9**] proposed that the corneal epithelium is renewed from a source of cells located at the limbus. They were the first in proposing the stem cell theory. Corneal stem cells are located peripherally at the limbus, in the basal cell layer, in pigmented crypts called the palisades of Vogt [**10**]. This pigmentation is thought to help protect the stem cells from ultraviolet light damage. In the normal cornea, the renewal occurs from basal cells with a centripetal migration of stem cells from the periphery. This is a structure deeply related to the function of each cell. The stem cells and their progenitors require the vascular nutrition that is found in the stromal vasculature outside the cornea, and thus they must be at the periphery [**2**].

Conversely, the cornea is an avascular structure. It must remain avascular in order to prevent vascular structures from interfering with light transmission and thus vision. The limbus plays an important role in preventing vascularization of the cornea from the conjunctiva; thus, with the loss of integrity of the limbus, conjunctival cells migrate to the cornea resulting in corneal neovascularization [**7**,**11**,**12**].

There are both primary and acquired causes of limbal stem cell deficiency in the cornea, which can be focal or diffuse, depending on the extent of limbal involvement with the underlying of the disease processes. The ocular diseases leading to LSCD can be subdivided in two major categories. The first category is characterized by the total destruction of the limbal stem cell population by chemical or thermal injuries, the Stevens-Johnson syndrome, multiple surgical or cryotherapy procedures in the limbal region, contact lens wearing [**7**,**13**,**14**], or severe microbial infection [**7**,**15**]. The second category includes diverse diseases such as aniridia [**7**,**16**], multiple endocrine deficiency, limbitis and peripheral ulcerative and inflammatory keratitis, neurotrophic and ischemic keratitis, and pterygium or pseudopterygium. These diseases do not destroy the limbal stem cells directly but instead damage the limbal stroma so that it cannot support these stem cells [**3**].

## Treatment

New developments made ocular surface reconstruction a widespread method in the treatment of severe ocular surface disease. A number of therapeutic strategies have been adopted to treat the limbal stem cell deficiency, by using several techniques with the same aim of restoring the stem cell function [**2**]. From the amniotic membrane transplantation, the limbal autograft and allograft, stem cells transplant and tissue engineering, they all contribute to the restoration of ocular surface integrity and vision. The limbal stem cell transplantation aims to replace the absent or damaged cells that are incapable of differentiating into the normal corneal epithelium, in order to regenerate the corneal- like epithelium. Stem cell therapy promotes re- epithelization, provides stable corneal epithelium, prevents regression of new vessels, and restores epithelial clarity. Tissue engineering is defined as the development of biological substitutes for the purpose of restoring, maintaining and improving tissue function and requires the application of principles and methods from both engineering and life sciences [**17**,**18**]. Scaffolds are developed to support the host cells during tissue engineering, promoting their differentiation and proliferation throughout their formation into a new tissue. The amniotic membrane can be used for several indications, either as a graft to replace the damaged ocular surface stromal matrix or as a patch (dressing) to prevent the unwanted inflammatory insults from gaining access to the damaged ocular surface, or a combination of both. The amniotic membrane facilitates the epithelization and reduction of inflammation, scarring, and vascularization. Compositionally, the basement membrane of the AM resembles that of the conjunctiva. The basement side of the membrane can act as a substrate for supporting the growth of the epithelial progenitor cells by prolonging their lifespan and maintaining their clonogenicity. This may support the idea of using the AM transplantation to expand the remaining limbal stem cells and corneal transient amplifying cells during the treatment of partial limbal deficiency [**2**,**19**] and to facilitate the epithelialization for persistent corneal epithelial defects with stromal ulceration [**2**,**20**–**22**]. In tissue cultures, AM supports the epithelial cell grown from the explant cultures [**2**,**23**–**25**] or other cultures [**2**,**26**,**27**], and maintains their normal epithelial morphology and differentiation. The AM can also be used to promote the non-goblet cell differentiation of the conjunctival epithelium [**24**]. The extent of limbal deficiency highlights the presence or absence of the central corneal transient amplifying cells and the depth of central corneal involvement. AMT is an important adjunct in limbal transplantation for both transplanted limbal stem cells to expand on the recipient eye and the residual stem cells to expand in the donor eye. Partial limbal deficiency can be reconstructed by AMT without the use of limbal transplantation [**28**]. However, for moderate unilateral or focal LSCD, the autograft limbal transplantation is the treatment of choice and for those with bilateral limbal deficiency, the treatment of choice is the allograft limbal transplantation, which invariably poses the challenge of allograft rejection. As an alternative to limbal grafting, corneal stem cell therapy may be considered for some patients, but the next stage in ocular surface reconstruction is the identification of corneal epithelial stem cells and the transplantation of bioengineered tissue, including the isolated stem cells.

## Conclusions

Science is continuously evolving. New era and new developments lead the way to new diseases, and more importantly, to new treatments. After years of clinical research and various theories, limbal stem cell deficiency diagnosis is now available and the treatment strategy applied.

After the suppression of inflammation, amniotic membrane transplantation can be performed in mild LSCD, keratolimbal allograft with amniotic membrane transplantation in moderate LSCD, and keratolimbal allograft or autograft with amniotic membrane transplantation and keratoplasty in severe LSCD. Perhaps time will improve the treatment strategy and stem cell transplantation and tissue engineering will become the most common treatment in ocular surface reconstruction for limbal stem cell deficiency.
